# Serum levels of a subset of cytokines show high interindividual variability and are not altered in rats transgenic for Huntington´s disease

**DOI:** 10.1371/currents.RRN1190

**Published:** 2010-10-25

**Authors:** Huu Phuc Nguyen, Maria Björkqvist, Felix J. Bode, Michael Stephan, Stephan von Hörsten

**Affiliations:** ^*^Department of Medical Genetics, University of Tuebingen; ^†^Lund University, Sweden; ^‡^Department of Neurology, University of Bonn, Sigmund-Freud-Str. 25, 53105 Bonn, Germany; ^§^Hannover Medical School and ^¶^Friedrich-Alexander-University Erlangen-Nürnberg

## Abstract

To evaluate whether cytokines are altered in peripheral blood of rats transgenic for the human Huntington´s disease mutation we investigated serum levels of GRO/KC, IL-1β, IL-13 and TNF-α at a symptomatic stage at 12 months of age. Overall serum levels of these cytokines were not significantly changed between transgenic HD rats and controls. Moreover, we observed a high interindividual variability. Our results indicate that these cytokines will be difficult to pursue as biomarkers in at least this rat model of HD.

****

## **Introduction**

Huntington's disease (HD) is an autosomal dominantly inherited neurodegenerative disease that is characterized by motor, psychiatric, and cognitive symptoms leading to progressive disability and eventually early death. Despite the underlying genetic defect being known for more than 15 years [1], there is currently no effective treatment. Previous studies have identified activated microglia in the brains of premanifest HD patients in vivo [2] and post mortem [3]. Furthermore, proteomic profiling of plasma from HD patients and healthy controls has demonstrated that many components of the innate immune system, such as clusterin and IL-6, were upregulated in HD patients [4]. Recently, Bjorkqvist and colleagues showed that inflammatory cytokines (especially IL-6, IL-8 and TNF-α) were elevated both centrally and peripherally in HD patients, and increased with disease progression [5]. Notably, interleukin 6 levels were increased in HD gene carriers with a mean of 16 years before the predicted onset of clinical symptoms. Similarly, serum levels of cytokines, measured by multiplex ELISA, were elevated in several mouse models of HD. We have generated a transgenic rat model of HD that has been investigated to a large extent. With a detailed evaluation of onset and time course of behavioral abnormalities in all three key systems affected in HD, in addition to a precise characterization of appearance and distribution of neuropathological markers, this animal model is highly suitable for testing therapeutic agents [6] [7]. Until the age of 6 to 9 months transgenic HD rats are considered as ‘presymptomatic’, whereas at the age of 9 months motor deficits and cognitive impairment are detectable marking the ‘symptomatic stage’. The bigger size of rats compared to mice also allows us to perform repetitive in vivo imaging such as PET and repetitive sampling of larger amounts of biofluids and therefore gives us a unique chance to follow up disease progression as well as effects of long-term treatment in vivo. To evaluate a possible increase of peripheral cytokine levels in the HD rat we investigated serum levels of GRO/KC, IL-1β, IL-13 and TNF-α in our transgenic HD rat model at the age of 12 months.

## **Material and Methods**

### **Animals**

Transgenic HD rats carrying a truncated huntingtin cDNA fragment with 51 CAG repeats under the control of the native rat huntingtin promoter, and their wildtype littermates were used. The expressed gene product was about 75 kDa, corresponding to 22% of the full-length huntingtin (cDNA position 324–2321, amino acid position 1-709/825, corresponding to exon 1-16), which are under the control of 886 bp of the rat huntingtin promoter (position 900 to 15). A colony of tg rats derived from a Sprague Dawley founder rat was established at the central animal facilities at Hannover Medical School, and the line was maintained by persistent inbreeding. For the experiments, fully inbred animals derived from generation F12 were used.

Genotyping was carried out as previously described [6] [7]. The number of CAG-repeats was analyzed in a subset of transgenic rats using these primers: cggctgaggcagcagcggctgt (forward) and ccttcgagtccctcaagtccttc (reverse). The reverse primer is labeled at the 5´end with the fluorescent dye Cy5. The PCR amplicon length was then analyzed on the Beckman coulter sequencer (CEQ 8000 Cycle Sequencer, Krefeld, Germany). In more than 15 generations we have observed very little variation of ±1-2 CAGs in transgenic rats of HD. Also for this study in a subset of animals the CAG size was checked and CAG length was found to be 51 ± 2 CAGs. After genotyping by qrtPCR, rats were housed in gender- and genotyped matched groups of two, according to FELASA recommendations. All rats were kept under a 12:12 hours light-dark cycle with lights on at 06.00 a.m. and food (Altromin lab chow pellets, Altromin standard diet: 1320; Lage, Germany) and tap water available ad libitum. All research and animal care procedures had been approved by the district government in Hannover, Germany, and followed principles described in the European Community’s Council Directive of 24th November, 1986 (86/609/EEC).

A set of 12 months old rats consisting of 20 homozygous transgenicHD rats (10 females and 10 males, respectively) and 20 wildtype littermates (10 females and 10 males, respectively) was used in this study.  Rats were deeply anesthetized with an isofluran-inhalation-anesthesia, and subsequently, the retrobulbar venous plexus was punctured with a glass capillary and approximately 1 ml blood taken. Blood drawing and handling was performed by two experimenters (F.B. and M.S.) on one day between 8 am and 11 am. All samples were immediately cooled to 4°C and centrifuged at 400 rpm for 20 min (Sigma 4K15, Osterode am Harz, Germany) and the supernatant was taken. Afterwards, all samples were stored at -80°C until measurement. Serum cytokine levels (GRO/KC, IL-1β, IL-13 and TNF-α) were quantified using Meso Scale Discovery (MSD®, Gaithersburg, MD) electrochemoluminescence multiplex assay using a modification of the manufacturer’s protocol. 20 ul was used as the sample volume and a 10-point standard curve was used, ranging from 2500 pg/ml to 0 pg/ml. The sample and calibrator were incubated on the MSD plate for 3 h (instead of 2 h), followed by a wash (as per manufacturer’s recommendation). The MSD plate was then incubated with detection antibody solution for 3 h (instead of 2h) before wash and read as per manufacturer’s recommendation).  Results were analyzed on a SECTOR™ 2400 instrument (MSD).

### **Statistical analysis**

Clear outliers that have values more than two standard deviations above the mean value were excluded from statistical analyses*. * Since gender differences have been reported for transgenic HD rats (Bode et al., 2008), we analyzed whether this also applies to the cytokine levels that we had investigated. One-way ANOVA for the factor sex as well as two-way ANOVA for the factors genotype and sex did not reveal any significant differences. Therefore, we had combined the results from both males and females for further statistical analyses. Data were then subjected to unpaired two-tailed t tests to identify significantly different serum levels for each cytokine. A critical value for significance of *p *< 0.05 was used throughout the study. All data represent means ± SEM.

## **Results**

Table 1 summarizes the results ofMultiplex ELISA quantification of cytokine levels in serum from transgenic HD rats (at the age of 12 months, symptomatic stage) compared with control animals. Notably, we observed that in a significant number of rats (both transgenic and wildtypes) serum levels of TNF-α, IL-13 and Il-1b were below the detection limit and a value of zero was recorded. Although a zero value (lack of evidence) cannot be considered as evidence for a lack of the cytokines investigated, this poses a statistical problem. Here we present data both including and excluding zero values (see table 1).  Nevertheless a large variability of cytokine levels in HD rats and controls was observed (Table 1) and no significant differences in absolute values between groups were found for any of the four cytokines investigated.

**Table 1: Serum levels of TNF-α, IL-13**, **IL-1β and GRO/KC in HD rats at the age of 12 months. NS: non significant, excl.: zero values excluded, incl.: zero values included**

**Table:** 

**Genotype**	**TNF-α**		**IL-13**		**IL-1β**		**GRO/KC**	
	**N**	**(ng/l) ± SEM**	**N**	**(ng/l) ± SEM**	**N**	**(ng/l) ± SEM**	**N**	**(ng/l) ± SEM**
**TG excl.**	12	33.91±6.547	12	2.192±0.6428	5	22.21±6.206	19	49.98±3.433
**WT excl.**	13	29.06±5.468	8	1.576±0.4485	6	24.50±5.239	20	44.50±5.060
**TG incl.**	18	21.43±5.601	20	1.315±0.4519	18	5.845±2.751	19	49.98±3.433
**WT incl.**	19	18.89±4.731	18	1.744±0.7386	19	7.737±3.101	20	44.50±5.060
**p value**	NS		NS		NS		NS	

Figure 1 illustrates the level of the various cytokines in transgenic HD rats in comparison to control levels (mean value of wildtype animals was set to 100%). There was no significant difference between transgenic HD rats and controls regarding serum levels of TNF-α or IL-1β, and although there was a trend towards higher serum levels of GRO/KC and IL-13 in transgenic animals compared to wildtype animals, this observation did not reach significance. This might be due to technical limitations or due to the large biological variability of these cytokines and it is in agreement with observations in mice and humans [8].

**Figure:**
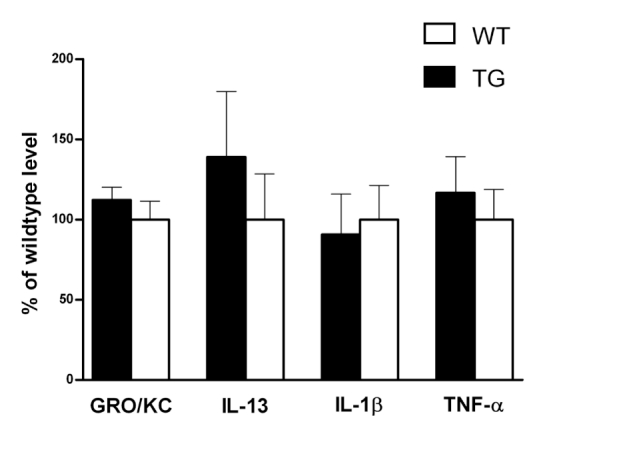
Figure 1 

## **Conclusion**

Taken together, the investigated pro-inflammatory cytokines were not significantly changed between transgenic HD rats and their wildtype littermate controls at the age of 12 months where motor deficits, cognitive impairment and neuropathological abnormalities are readily detectable. Our results indicate that there is a significant biological variance in serum levels of these cytokines and that these cytokines will be difficult to pursue as biomarkers in at least this rat model of HD. However, we only investigated a subset of cytokines, and notably, IL-6 was not measured.

## **Funding**

European Community ‘RATstream STREP project’ (037846 to H. Nguyen and S. von Hörsten)

****

## **Competing interests**

The authors have declared that no competing interests exist.
